# Mycotoxins in Fish Aquaculture—Occurrence and Future Perspective

**DOI:** 10.3390/foods14244301

**Published:** 2025-12-14

**Authors:** Ana Vulić, Nina Kudumija, Tanja Šegvić-Bubić, Tina Lešić

**Affiliations:** 1 Laboratory for Analytical Chemistry, Croatian Veterinary Institute, Savska Cesta 143, 10000 Zagreb, Croatia; vulic@veinst.hr (A.V.); lesic@veinst.hr (T.L.); 2 Laboratory for Aquaculture, Institute of Oceanography and Fisheries, Šetalište Ivana Meštrovića 63, 21000 Split, Croatia; tanja.segvic@izor.hr

**Keywords:** toxic fungal metabolites, farmed fish, feed contamination, tissue residues

## Abstract

Mycotoxins are toxic secondary metabolites produced by filamentous fungi which are commonly found as natural contaminants in food and feed worldwide. In recent years, aquaculture research has increasingly focused on changing fish feed by replacing traditional protein sources with plant-based and insect ingredients to promote sustainability. However, this shift has raised concerns about mycotoxin contamination in farmed fish, fish products, and processing by-products. As a result, the pursuit of sustainable aquaculture practices may inadvertently increase the risk of mycotoxin exposure. To date, studies on freshwater fish have focused primarily on regulated mycotoxins, and the findings have demonstrated their occurrence in muscle, liver, intestine, ovaries, and hepatopancreas. Most studies have investigated aflatoxin B1, and its presence has been confirmed in the muscle tissue of several fish species. In marine fish, research has encompassed a broader spectrum of mycotoxins, including emerging and masked forms, across multiple tissues and organs. However, across various studies, conflicting results have been reported regarding the occurrence of emerging mycotoxins, particularly enniatins and beauvericin. This paper reviews current research on mycotoxin contamination in farmed fish, summarising detected levels across freshwater and marine species and in derived products, and also discusses future perspectives on mycotoxin risks in sustainable aquaculture.

## 1. Introduction

Mycotoxins are compounds produced by filamentous fungi, commonly detected as natural contaminants of agricultural commodities worldwide. They are considered secondary metabolic products that are not essential for mould growth and development [1,2]. Mycotoxins form a large group of organic compounds that differ significantly in structure and functionality. As they are toxic, exposure to mycotoxins through food or feed, even at low concentrations, can lead to mycotoxicosis in both animals and humans [3]. Mycotoxins are climate-dependent and associated with plants and storage conditions, but their presence is also affected by non-infectious factors such as the bioavailability of micronutrients, insect damage, and other pest attacks [4]. In food of plant origin, primary contamination with mycotoxins occurs when mycotoxin-producing moulds colonise a foodstuff under favourable conditions (suitable relative humidity, temperature, aeration, presence of insects, and physical damage to the substrate). Mycotoxins of major importance for human and animal health are produced by the following fungal genera: *Aspergillus* (produces aflatoxins (AFs) and ochratoxin A (OTA)), *Fusarium* (produces fumonisins (FUMs), zearalenone (ZEN), deoxynivalenol (DON), and trichothecenes), *Penicillium* (produces OTA), and *Alternaria* (produces Alternaria toxins). Aflatoxin B1 (AFB1) has been classified as a human carcinogen (Group 1), OTA and fumonisins as possible human carcinogens (Group 2), while certain trichothecenes (TCNs), ZEN, and alternariol (AOH) are deemed non-carcinogenic (Group 3) but can also cause health disorders, including suppression of the immune response (TCNs), reproductive disorders (ZEN), and disruption of endocrine response (AOH) [5]. In recent decades, global mycotoxin contamination has increased, largely driven by climate change [6,7,8,9]. In addition to well-known mycotoxins, numerous modified or emerging forms are increasingly reported. Our recent study demonstrated that several modified mycotoxins in maize, wheat, triticale, barley, and oat can reach concentrations of up to 10 mg/kg [10,11]. Worldwide, 60–80% of major crops contain detectable mycotoxins, and 20–25% exceed EU or Codex Alimentarius limits for regulated mycotoxins in food and feed [12]. Long-term monitoring of cereals grown in Croatia shows an increasing trend of contamination with Fusarium mycotoxins, including FUMs, ZEN, DON, T-2, and HT-2 toxins [6], consistent with patterns observed not only in Europe but worldwide. Increasing attention has also been directed to emerging and masked mycotoxins [13,14]. These new mycotoxins are categorised as emerging mycotoxins and are described as “mycotoxins that are not regularly detected or controlled by legislation, but the evidence of their occurrence is quickly growing” [15]. In addition to the above-mentioned mycotoxins, masked mycotoxins became an important subject of investigation in the early 1990s, as they could not be determined by conventional analytical methods [16]. They are formed either as a result of the plant’s defence mechanism against mould infestation—by altering the chemical structure of the mycotoxin—or by fungi, bacteria, or animals. In contrast to their parent compounds, masked mycotoxins are often less toxic, but after digestion they become bioavailable and harder to detect,; thus, they represent a hidden danger in the food chain [17]. Some characterised modified forms of mycotoxins formed by fungi include 3- and 15-acetyl-DON and ZEN-14-sulphate; by plants, 3-O-glucoside-DON, ZEN-14-glucoside, and ZEN-16-glucoside; by animals, DON-3- and DON-15-glucuronide and DON-sulphonates; and by bacteria, deepoxy-DON and DOM-1. These compounds are often not routinely analysed and therefore not reported [17,18,19,20]. As regulatory limits for these modified forms have not been established, it remains unclear whether grains contaminated with such mycotoxins are safe for animal or human consumption. Therefore, research in the field of emerging and masked mycotoxins should focus on broadening the range of mycotoxins routinely tested, the types of food and feed tested, and collecting data on their occurrence. Based on these data, future regulatory limits and risk assessments for humans and animals could be performed.

### 1.1. Mycotoxins in Aquafeeds

Data on the occurrence of mycotoxins in aquaculture and the risks they pose to the sector remain limited. Commercial fish feeds typically include plant-derived ingredients such as soybean meal and cereal grains, which may serve as significant sources of contamination [20,21]. Replacing fishmeal with plant proteins increases exposure risk. For example, wheat inclusion ranges from 15 to 27% in rainbow trout (*Oncorhynchus mykiss*) complete feed mixtures, and from 20 to 70% in cyprinid feeds [22]. As wheat is often contaminated with mycotoxins, it poses an increasing risk of exposing fish to these toxins. Mycotoxin-contaminated fish feed is a widespread problem. In many developing regions, especially in tropical areas, feed is produced under suboptimal milling and storage conditions, exacerbating contamination [23,24]. Within the EU, maximum levels for mycotoxins in fish feed are not regulated, apart from a recommended limit of 10,000 µg/kg for fumonisins in aquaculture feed [25]. Recent studies into mycotoxin occurrence in fish feed have shown that the most prevalent compounds are produced by species of the *Fusarium* genus. Marín-Sáez et al. [26] reported that ENNB and ENNB1 were detected in 81% of analysed samples, with concentrations ranging from 1.54 to 13.5 μg/kg. Additional mycotoxins identified included ZEN (19%); alternariol (ALT), alternariol monomethyl ether (ALT MOME), beauvericin (BEA) (14%); AFB1 (11%); DON (8%); ENNA and OTA (6%). Although present in a smaller proportion of samples, ZEN, DON, and OTA exhibited the highest concentrations, reaching up to 135, 63, and 202 μg/kg, respectively. Comparable results were described by Koltesi et al. [27], who also identified *Fusarium*-derived toxins as the most prevalent: fusaric acid (FA, 55%), DON (48%), FUMB1 (36%), FUMB2 (27%), and the masked mycotoxin DON-3-Glc (18%). All feed samples analysed (*n* = 44) were contaminated with at least one mycotoxin, and 75% contained multiple mycotoxins concurrently. Generally, these findings reinforce the widely reported observation that animal feed samples frequently contain several mycotoxins (75–100%), particularly when diets include more than one plant-derived ingredient.

### 1.2. Feed Sustainability and New Protein Sources

Global aquaculture production increased four-fold between 1995 and 2015, rising from 12.2 million tonnes to 50.7 million tonnes, which drove a parallel six-fold increase in aquafeed production, from 7.6 million tonnes to 47.7 million tonnes. Although aquafeeds account for less than 4% of total global feed production, their ingredients overlap with those used in terrestrial livestock, pet food, and human food sectors. Identifying alternative and sustainable protein sources is therefore a priority to ensure a socially and environmentally sustainable industry [28]. Historically, fish feed was mainly produced from fish meal and fish oil. Today, aquaculture still relies heavily on these ingredients, which are sourced from wild-captured forage fish. However, increasing the use of forage fish is unsustainable, especially given the estimation that an additional 37.4 million tonnes of fish feed will be required by 2025 [29]. This growing demand highlights the urgent need for alternative protein sources. Over the past decade, aquaculture research has focused on incorporating plant-based ingredients into fish diets as a primary protein source. In addition to plant-based options, fishery and aquaculture by-products, as well as insect meals, have emerged as promising candidates to meet the protein requirements of aquaculture feeds over the next 10–20 years [28]. In particular, insects are attracting significant interest as a sustainable and efficient alternative protein source; however, information on mycotoxin contamination in insects remains limited [30]. Most studies addressing mycotoxin accumulation in insects, specifically *Hermetia illucens*, *Tenebrio molitor*, and *Alphitobius diaperinus*, indicated that mycotoxin residues are below the applied method’s limit of detection or limit of quantification. These studies focused on regulated mycotoxins, including AFB1, DON, OTA, ZEN, FUMB1, FUMB2, T-2, and HT-2 toxin. As data from these studies cannot be generalised across species or even across strains of the same species, further studies are recommended [31]. In addition to insect exposure to mycotoxins via feed, poor manufacturing practices can also lead to contamination of the substrate used for insect rearing (such as straw); however, contamination of larvae as final products was not found [32]. Although this is promising regarding mycotoxin contamination, there is a lack of studies addressing the occurrence of masked or modified mycotoxins in insects. Unfortunately, these new feed formulations, which use insects as a protein source and are tested to meet sustainability requirements, can unintentionally introduce mycotoxins into aquaculture production. Two major concerns arise: (i) the introduction of mycotoxins and their modified forms into fish tissues, with potential implications for both fish and human health; and (ii) the recirculation of mycotoxins within the production chain, particularly if fish by-products from fisheries and aquaculture are used. Fish aquaculture by-products include skeleton, head, scales, skin, and viscera [33], which are used in feed industry for the preparation of fish flour and fish oil. In the case of mycotoxin contamination, especially with chemically stable ones, the circulation in fish food chain can be expected. To date, to the best of our knowledge, there are no studies that have assessed the stability of mycotoxins during the preparation of fish flour from contaminated by-products.

### 1.3. Routes of Mycotoxin Contamination in Aquaculture

**Figure 1:**
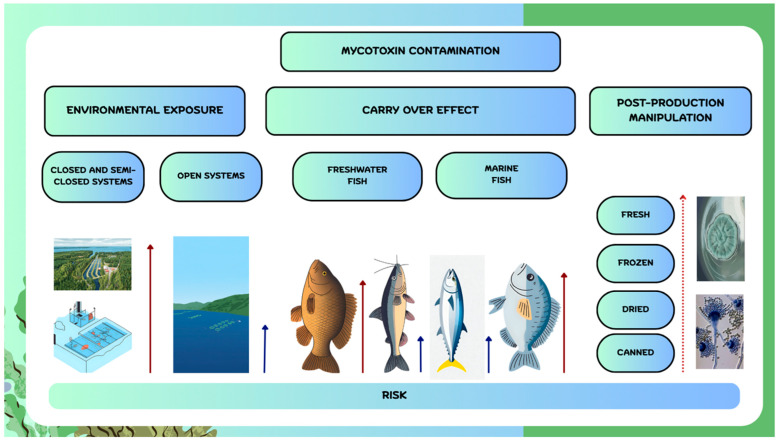
Routes of mycotoxin contamination in fish aquaculture and fish products. (Red arrows—higher risk; blue arrows—lower risk; dotted arrow—increasing risk).

Mycotoxin contamination in aquaculture products may occur through (i) environmental exposure, (ii) carry-over from feed, and (iii) post-production handling (Figure 1). Environmental contamination can result from agricultural runoff, wastewater, or improper disposal of contaminated materials. Although their stability in water varies, some mycotoxins, including DON and ZEN, can persist in the environment, potentially posing a risk to water sources such as lakes, rivers, and groundwater [34]. Bucheli et al. [35] reported the presence of ZEN and DON in Swiss rivers, with concentrations of up to 4.9 µg/L for DON and 35 ng/L for ZEN. To the best of our knowledge, the potential for mycotoxins to contaminate water—particularly considering water-stable compounds and closed or semi-closed aquaculture systems—has not yet been investigated. This route of contamination is less likely, especially in marine sea-cage aquaculture, which is conducted in open-water systems. Future research should be implemented to assess the rate of dermal and mucosa transfer of mycotoxins from water to fish, which is currently a research gap. Feed remains the primary contamination pathway. Herbivorous freshwater species consuming cereal-based diets are most vulnerable, whereas carnivorous freshwater fish relying on animal proteins face lower risk. In marine aquaculture, production largely centres on carnivorous species; however, sustainability goals—particularly the reduction or replacement of fish meal and fish oil with plant-based ingredients—have increased the risk of mycotoxin contamination in formulated feeds. This contamination route, as it will be discussed in further sections, was studied on several freshwater and marine fish species. Due to the limited number of samples per study, different way of feed contamination (natural or fortified), different levels of contamination, different analytical methods used for mycotoxin quantification unambiguous conclusion about mycotoxin occurrence could not be drawn. These scarce data are not enough to perform a human exposure study and risk assessment. Post-production contamination can result from inadequate sanitation during handling, processing, or packaging, beginning with catching, evisceration, transportation, and chilling or freezing. Mould spores are ubiquitous in soil, water, air, and on equipment, and can contaminate fish or fish products through contact [36]. Among different fish products, the highest risk is associated with fresh products, followed by frozen, dried, and finally canned products, which are processed at high temperatures. In general, when applying a good manufacturing practice which includes monitoring of critical points important for mould growth the risk of contamination of fish products could be minimised. Currently, the maximum levels (MLs) for mycotoxins in fish products have not yet been regulated within the EU.

## 2. Methods

This review was conducted as a narrative synthesis of the current scientific literature addressing the occurrence of mycotoxins in fish aquaculture. The literature was searched using PubMed, Scopus, Web of Science, ScienceDirect, and Google Scholar between September and October 2025. The search strategy combined broad and targeted terms related to mycotoxins, aquaculture, and food safety, including keywords such as *mycotoxins*, *aflatoxin*, *ochratoxin*, *Fusarium toxins*, *deoxynivalenol*, *fumonisin*, *zearalenone*, *T-2 toxin*, *emerging mycotoxins*, *enniatin*, *beauvericin*, and *contamination*, as well as *farmed fish*, *aquaculture*, and *freshwater* or *marine fish*, *fish products* using Boolean operators AND and OR, with general search strings (e.g., “mycotoxin” OR “fungal toxin”) AND (“aquaculture” OR “farmed fish”) applied alongside specific toxin-focused queries for greater precision (e.g., “aflatoxin *” AND “fish” OR “aquaculture”). The search was primarily focused on the recent literature available in English; however, earlier publications were also considered when they provided foundational or highly relevant information on mycotoxin occurrence in aquaculture systems. Studies were included if they reported mycotoxin contamination in farmed fish or fish products. Publications unrelated to aquaculture or mycotoxins, non-peer-reviewed sources, conference abstracts without data, and duplicates were excluded. Relevant articles were screened by title and abstract, reviewed in full, and synthesised qualitatively. Findings were organised around key themes, including mycotoxin occurrence in freshwater and marine species, contamination of fish products.

## 3. Mycotoxin Contamination in Freshwater Fish

Freshwater fish are widely consumed due to their high nutritional value, particularly their abundant protein content, favourable fatty acid composition, and richness in micronutrients [24,37]. Alongside marine species, freshwater fish represent a major component of global aquaculture production, and their consumption continues to increase worldwide. These fish are typically fed commercially prepared plant-based diets containing varying proportions of cereals, which can act as significant sources of mycotoxins. Consequently, the presence of mycotoxins in feed can lead to their carry-over into edible fish tissues, as demonstrated in several studies [38,39,40].

Although the impact of mycotoxins in aquaculture systems has not been sufficiently investigated, several authors have documented toxic effects in fish comparable to those observed in more extensively studied terrestrial livestock species such as pigs and poultry. These include liver and kidney diseases and suppression of the immune system [24]. For example, Matejová et al. [38] identified rainbow trout as a highly sensitive fish species to AFB1, which has proven carcinogenic and hepatotoxic effects. Earlier studies have also reported the toxic effects of AFB1 in catfish (*Clarias gariepinus*) and Nile tilapia (*Oreochromis niloticus*) [41,42]. Both acute and chronic toxicity have been demonstrated in rohu (*Labeo rohita*), manifesting as reduced food intake, sluggish movement, and pathological changes in the liver, spleen, intestines, pancreas, and gills [43]. In contrast, [44] state that gibel carp (*Carassius auratus gibelio*) is relatively less sensitive to AFB1 at dietary concentrations up to approximately 1000 μg/kg over 12 weeks, and demonstrated an effective ability to eliminate AFB1 during a recovery period on uncontaminated feed.

Due to the increasing consumption of freshwater fish, Sun et al. [45] developed a multimethod for mycotoxin determination in fresh fish (muscle tissue and offal) and dried seafood. The results showed that ZEN and OTA were the most prevalent mycotoxins, occurring in 29.6% and 14.8% of the samples, respectively. Trace amounts of AFB2 were detected in carp (*Carassius carassius*) muscle tissue, while DON, T2, and HT-2 toxins were not detected in any samples analysed. In a 72-day exposure study to feed containing 2 mg/kg ZEN, Woźny et al. [46] found no ZEN residues in rainbow trout muscle tissue, although low concentrations (~2 µg/kg) were present in the liver and intestines, and higher levels (7.1 ± 3.2 µg/kg) were detected in the ovaries. While these concentrations do not pose a direct risk to human health, ZEN exhibited anabolic effects in fish, which the authors linked to impaired immune function. Similarly, Pietsch et al. [47] detected low ZEN concentrations (0.13–0.22 μg/kg) in carp muscle after exposure to dietary ZEN using different concentrations, but observed no oestrogenic effects, likely due to rapid metabolism of ZEN in carp. Furthermore, Woźny et al. [48] also reported negative effects of ZEN on the reproductive outcomes of rainbow trout, noting that fish undergoing sexual maturation may be particularly sensitive to ZEN contamination in food. Aflatoxin metabolism in fish involves the reduction of AFB1 to aflatoxicol (AFL), which can be excreted in bile [39]. Nevertheless, carry-over into edible tissues has also been reported in freshwater species exposed to AFB1-contaminated feed. Residues have been detected in muscle and liver of Nile tilapia [49,50,51] and in catfish [52]. Gomes et al. [53] reported the presence of AFB1 in 70% of liver samples and 43.3% of muscle samples from several commonly consumed roundfish species in Brazil, including tambaqui (*Colossoma macropomum*), pirapitinga (*Piaractus brachypomus*) and pacu (*Piaractus mesopotamicus*). The maximum concentrations detected were 5.7 µg/kg in liver and 1.13 µg/kg in muscle. Although AFB1 levels in catfish muscle were low [52], growth performance was clearly impaired, particularly at higher levels of dietary contamination. Significant losses in aquaculture caused by aflatoxin exposure have also been reported elsewhere [49]. In sturgeon (*Acipenser ruthenus*), AFB1 accumulation in muscle and liver was strongly dose-dependent, increasing linearly with dietary AFB1 concentration [54]. In a market-based survey from Qatar, AFs were detected in one liver sample and in two muscle samples of rohu (*Labeo rohita*), with mean concentrations of 3.86 μg/kg and 1.54 ± 0.12 μg/kg, respectively. OTA was not detected in the muscle samples; it was found only in one liver sample at a concentration of 1.30 μg/kg [37].

A summary of studies reporting the occurrence of mycotoxins in freshwater fish species is presented in Table 1.

Although research on the prevalence and impact of mycotoxins in freshwater aquaculture is growing, available data remain limited. This is due to the large number of fish species, variability in exposure duration, environmental factors on farms, and the influence of climate change on fungal growth and toxin production.

Table 1 shows that most results pertain to freshwater fish kept under experimental conditions and fed diets enriched with specific mycotoxins, while only a few references address the examination of the carry-over effect in cases of natural mycotoxin contamination of feed to freshwater fish from farms or from the market. Moreover, in most experimental designs, feed fortification involved the addition of a single mycotoxin, whereas naturally contaminated feed typically contains multiple mycotoxins occurring simultaneously in various combinations. This is attributable to the incidence of multimycotoxin contamination in cereals used for feed production [10,11].

When comparing the analytical methods used to detect mycotoxins, HPLC methods with UV (ultraviolet), diode array (DAD) and mass detectors are predominant compared to the ELISA (enzyme-linked immunosorbent assay) technique. Based on this, it can be concluded that sophisticated analytical techniques, which are capable of providing unambiguous results, are increasingly replacing ELISA methods, which may yield false-positive results due to the cross-reactivity of structurally similar mycotoxins. Furthermore, when comparing the occurrence of mycotoxins in freshwater fish tissues and organs, most studies have focused on AFB1 due to its well-documented high toxicity, followed by a few studies on other regulated mycotoxins (ZEN, DON, FUMs, T-2, and HT-2). However, none of the studies included either emerging or masked/modified mycotoxins.

The most frequently studied matrices in freshwater fish are muscle and liver. A carryover effect of AFB1 to muscle was confirmed in all experiments, regardless of fish species. The AFB1 concentrations detected in muscle were highly variable, ranging from 0.05 to 78 µg/kg, reflecting differences in administered doses and target species. The only two comparable studies [49,50], both conducted on Nile tilapia with similar AFB1 doses of 200 and 250 µg/kg and using the same detection technique, nevertheless produced markedly different results, ranging from 3.7 µg/kg to 78.33 µg/kg. The two studies on ZEN carryover conducted on Common carp and Carp used different types of contaminated feed (naturally contaminated vs. spiked), making direct comparison of the results questionable, although a carryover effect was observed in both cases.

Overall, the available studies on mycotoxin carryover in freshwater fish are limited in scope, rely predominantly on experimentally spiked feeds with single mycotoxins, and employ advanced analytical methods. Although carryover has been documented—particularly for AFB1 and ZEN—the variability of results, scarcity of data from naturally contaminated feeds, and absence of studies on emerging or masked mycotoxins highlight significant knowledge gaps that warrant further investigation.

## 4. Mycotoxin Contamination in Marine Fish

There is limited information on mycotoxin occurrence in aquacultured marine fish compared to livestock species but also to freshwater fish. While EFSA has evaluated mycotoxin carry-over in terrestrial animals such as poultry, swine, and cattle, data for farmed fish remain scarce [59].

The mycotoxins of greatest concern in animal-derived products include aflatoxins (AFs: B1, B2, G1, G2, M1), OTA, and *Fusarium* toxins, including FUMs (FB1, FB2, FB3), trichothecenes (DON, T-2, HT-2), and ZEN. Across studies on fish, as in general, AFs have been the most frequently investigated mycotoxins [24,37,60]. More recent research has also focused on emerging mycotoxins [39,61,62]. Most research on mycotoxins in aquaculture has focused on freshwater fish, reflecting their higher exposure to cereal-based feeds prone to fungal contamination. Species-specific differences in mycotoxin metabolism further influence toxin distribution, where edible muscle may contain metabolites rather than parent compounds [63].

A summary of studies reporting the occurrence of mycotoxins in marine fish species and derived products is presented in Table 2.

Several studies have investigated the occurrence and carry-over of regulated mycotoxins, including AFB1, OTA, and *Fusarium* mycotoxins, in marine fish. El Sayed and Khalil [60] reported that European sea bass is highly sensitive to AFB1, detecting up to 4.25 µg/kg in edible muscle after exposure to 0.018 mg/kg body weight AFB1 for 42 days. The observed carry-over of AFB1 in European sea bass muscle exceeded levels typically reported for terrestrial livestock. Similarly, controlled feeding experiments in Atlantic salmon demonstrated a tissue-specific distribution of mycotoxins. Bernhoft et al. [69] supplemented Atlantic salmon diets with 2 or 6 mg/kg DON and 0.8 or 2.4 mg/kg pure OTA for up to eight weeks and found that DON was evenly distributed across liver, kidney, muscle, skin, and brain, reaching up to 28.6 µg/kg in liver and 18.6 µg/kg in muscle. In contrast, OTA was mainly confined to liver and kidney, with only trace or non-detectable concentrations in other tissues (maximum 4.81 µg/kg in liver). These findings, consistent with Pietsch (2019) [72], suggest that fish are particularly sensitive to OTA, which tends to concentrate in hepatic and renal tissues. Higher OTA concentrations observed in the kidney and liver are consistent with previous studies and likely reflect tissue-specific reabsorption and distribution mechanisms, as well as the activation of hepatic elimination pathways, which together limit OTA accumulation in muscle [60,62,65,69]

Further carry-over investigations support the notion that transfer of regulated mycotoxins from feed to edible tissues is generally limited under aquaculture conditions. Nácher-Mestre et al. [71] reported that gilthead sea bream and Atlantic salmon fillets contained no detectable mycotoxins after seven to eight months of feeding with diets naturally contaminated mainly with DON (19.4–79.2 µg/kg) and FBs (6.4–754 µg/kg). Similarly, Johny et al. [73] found no mycotoxin residues in Atlantic salmon when feed concentrations were below the limit of quantification (<LOQ).

Naturally occurring contamination, however, has been reported in market fish. AFs were found in liver samples of marine species from Qatar (up to 2 µg/kg), though OTA was not detected. However, the limited number of analysed samples prevented definitive conclusions [37]. No OTA was detected in 60 fish muscle samples analysed between 2003 and 2012 under the National Residue Control Programme [74]. Conversely, naturally occurring OTA was found in muscle, liver, and kidney of gilthead sea bream and European sea bass, sold in Italian markets, with higher concentrations detected in non-edible tissues (0.91 µg/kg) compared with muscle (0.28 µg/kg) as already stated, without significant differences between the two species [65].

Emerging *Fusarium* mycotoxins, particularly ENNs and BEA, have also been investigated in farmed fish from Spanish markets, including European sea bass, gilthead sea bream, and Atlantic salmon. In market samples of European sea bass and gilthead sea bream, ENB was consistently the most prevalent mycotoxin, whereas ENA and BEA were generally not detected. ENA1, ENB1, and ENB were found in all analysed tissue types, with concentration up to 45.0, 119.0, and 72.3 µg/kg, respectively, and ENA detected only in liver at 84 µg/kg [62]. Although the proportion of positive samples in fish tissues was lower than in feed, the mycotoxin concentrations in tissues were higher, suggesting that tissue levels are influenced by the timing of toxin withdrawal from the diet. Earlier studies on sea bass and sea bream reported similar findings, with ENB detected in muscle and liver at concentration up to 44.7 µg/kg, while ENA and BEA were not detected [63]. Subsequent research demonstrated higher ENs levels in Atlantic salmon compared with European sea bass, gilthead sea bream, and rainbow trout, with concentrations ranging from 22 to 103 µg/kg. This difference is likely attributable to the lipophilic nature of ENs and the higher lipid content of Atlantic salmon (7.4 g/100 g edible portion) compared with European sea bass (1.3 g/100 g) [70]. The detection of ENNs in marine aquaculture supports two hypotheses: first, that other *Fusarium* mycotoxins, such as FUMs, DON and ZEN, may be present at higher concentrations but were not analysed; and second, that ENNs may be preferentially deposited in muscle tissue relative to DON or FUM, even when present at lower concentrations in feed, consistent with their frequent co-occurrence with major *Fusarium* mycotoxins [40]. In contrast, [61] reported no carry-over of ENNs or BEA from feed spiked with ENB (19.9 µg/kg) and BEA (30 µg/kg) to gilthead sea bream and Atlantic salmon, indicating that bioaccumulation may depend on species-specific factors and feed composition.

### Mycotoxin Contamination in Marine Fish Products

Unlike fresh fish, processed products undergo preservation such as salting, smoking, or drying. While these methods extend shelf life, they do not fully prevent microbial growth, and therefore compromise product quality [64]. Mycotoxin contamination can occur during processing via airborne fungal spores, during improper storage under conditions of high temperature and humidity, or through handling at markets. *Aspergillus* and *Penicillium* are the most commonly associated genera [66], while contamination may also arise earlier in the production chain through mycotoxin-contaminated feed or later during storage [45].

Recent studies have further characterised the fungal communities and mycotoxin profiles in processed and dried fish products. *Aspergillus* species are most frequently reported in smoked fish, followed by *Penicillium* species, both of which are capable of producing a wide range of mycotoxins [75,76]. In recent study, however, *Fusarium* species have been identified as the predominant genus (80.4%), followed by *Penicillium* (70.7%) and *Aspergillus* (63.9%) [64].

Tolosa et al. [67] analysed 15 mycotoxins in smoked and sushi salmon products, and found no detectable levels in nearly 60 samples. Only trace amounts of FUS-X and ENB were found in certain surimi-based products, likely due to non-fish ingredients. These results indicate that adequate processing and proper storage conditions can effectively prevent fungal growth and mycotoxin formation in fish products. Consistent with this, Carballo et al. [68] reported that none of the 27 mycotoxins screened were detected in ready-to-eat marine fish meals.

In contrast, detectable mycotoxin levels have been observed in dried fish products. In a survey of six dried fish species marketed in China, 17% of samples contained at least one mycotoxin (AFB1, OTA, T-2 or DON) with AFB1 and OTA being the most prevalent and reaching concentrations up to 3.52 µg/kg for AFB1 [64]. All fish species included in the study showed a comparable degree of contamination. The study also included mould analysis, revealing Aspergillus flavus, a known producer of AFB1, as the most frequently isolated species. An earlier study by Deng et al. [66] similarly detected in 58% of dried sea fish sample analysed, most frequently AFB1, OTA, and occasionally T-2 toxin, with concentrations up to 1.51 µg/kg for OTA. Sun et al. [45] also reported OTA and ZEN in fish products from Chinese markets, with ZEN showing the highest occurrence and concentration (up to 317.3 µg/kg), while AFs, HT-2 and T-2 toxins, and DON were not detected. Although mycotoxin concentrations in dried seafood are generally low, their occurrence frequency is high. Therefore, regular monitoring of mycotoxin residues in dried sea-food, particularly in hot and humid climates, remains an important food-safety measure.

Although studies on marine fish remain limited, most research conducted over the past decade has focused on species such as gilthead sea bream (*Sparus aurata*), European sea bass (*Dicentrarchus labrax*), and Atlantic salmon (*Salmo salar*), as well as on dried marine fish products. Sea bass and sea bream display broadly similar physiological and metabolic characteristics, including comparable feeding habits and digestive physiology, which may lead to similar patterns of mycotoxin uptake and biotransformation compared to salmon. The detected mycotoxins in these studies mostly include AFB1, OTA, and several emerging mycotoxins such as ENNs, while data on masked/modified mycotoxins are still lacking. This lack of data on the presence of masked/modified mycotoxins in marine fish can be attributed to the limited testing of raw materials used in fish-feed analysis, as well as to the scarcity of analytical methods capable of detecting these mycotoxins in fish organs and muscle tissue.

Given that the studies mentioned are mostly from the past decade and have pre-dominantly used chromatographic methods rather than earlier ELISA-based approaches (as indicated in only one survey), it can be considered that the applied analytical techniques provide reliably low detection limits and that the reported mycotoxin levels in re-cent research can be regarded as analytically dependable.

Detected mycotoxin levels in fish, however, cannot be directly compared with regulatory thresholds, as no such limits currently exist in the European Union for these commodities. The most comparable guideline is provided by Italy, which has established a maximum level of 1 µg/kg OTA for pork meat and meat products [77]. Although this limit does not apply to fish, it provides a useful benchmark. When compared with this value, three out of eight studies reporting OTA in fish fillets detected concentrations exceeding this threshold, reaching around 2 µg/kg [45,64,66]. For marine fish, most available data concern naturally occurring mycotoxin residues have been identified through market-based surveys, particularly as nearly one-third of referenced studies mentioned focused on commercial fish products.

## 5. Conclusions

With the increase in aquaculture production and the shift towards more sustainable feed formulations, concerns regarding mycotoxin contamination in farmed fish, fish products, and by-products are growing. Mycotoxin contamination, primarily with AFB1 and, to a lesser extent, Fusarium mycotoxins, has been investigated mostly in freshwater fish, with fewer studies in marine species. However, data on emerging and masked mycotoxins in aquaculture systems remain scarce. Due to the limited number of studies and often low sample sizes, no human exposure or risk assessment specific to mycotoxins in farmed fish has been conducted to date. Future research should address these knowledge gaps, with particular emphasis on widely farmed species and on mycotoxins that show increasing trends in occurrence and co-occurrence. This includes both regulated and emerging mycotoxins, as well as their modified forms, in edible tissues and in aquaculture by-products destined for fish meal production.

## Figures and Tables

**Table 1:** Mycotoxin contamination in freshwater fish.

Mycotoxin	Fish Species	Feed Contamination/Fish Origin	Tissue/Organ	Contamination Level (µg/kg)	Detection Method	Reference
AFB1	Gibel carp (*Carassius auratus gibelio*)	Spiked/animal trail	Muscle, hepatopancreas (n = 25)	2.4 11.8	ELISA	[44]
AFB1	Juvenile gibel carp (*Carassius auratus gibelio*)	Spiked/animal trail	Muscle (n = 23)	4.08	ELISA	[55]
OTA, AFB2, ZEN, DON, T-2, HT-2	Carp (*Cyprinus carpio*) Crucian carp (*Carassius carassius*)	Natural/fish from market	Muscle, (n = 7) Entails (n = 11)	OTA: 0.5–14, AFB2: 1.2, ZEN: 11.2–14.8, DON: n.d., T-2: n.d., HT-2: n.d. OTA: 0.8, AFB2: n.d., ZEN: 0.5, DON: n.d., T-2: n.d., HT-2: n.d.	LC-MS/MS	[45]
ZEN	Rainbow trout (*Oncorhynchus mykiss*)	Natural/fish farms	Muscle, liver, intestine, ovaries (n = 9)	n.d. n.d. ≈2 ≈2–7.1	HPLC-FLD	[46]
AFB1	African sharptooth catfish (*Clarias gariepinus*)	Spiked/animal trail	Muscle (n = 270)	0.05–0.12	ELISA	[52]
AFB1	Hybrid sturgeon (*Acipenser* *ruthenus*)	Spiked/animal trail	Muscle, liver (n = 210)	≈5–34 ≈20–143	ELISA	[54]
AFB1	Lambari fish (*Astyanax* *altiparanae*)	Spiked/animal trail	Muscle, liver (n = 50 fish/m^2^)	19–50 243–265	HPLC-FLD	[56]
AFB1	Nile tilapia (*Oreochromis niloticus*)	Spiked/animal trail	Muscle, liver (n = 240)	3.7 5.0	HPLC-FLD	[49]
AFB1	Nile tilapia (*Oreochromis niloticus*)	Spiked/animal trail	Muscle (n = 360)	78.33	HPLC-FLD	[50]
AFB1	Nile tilapia (*Oreochromis niloticus*)	Spiked/animal trail	Muscle (n = 360)	1.87	HPLC-FLD	[51]
DON	Grass carp (*Ctenopharyngodon* *idella*)	Spiked/animal trail	Intestine (n = 1440)	17.64–28.82	HPLC-FLD	[57]
ZEN	Common carp (*Cyprinus carpio* L.)	Spiked/animal trail	Muscle (n = 72)	ZEN: 0.13–0.22, α-ZEL: 0.11–0.16	HPLC-FLD	[47]
STG	Nile tilapia (*Oreochromis niloticus*)	Spiked/animal trail	Muscle (n = 40)	0.9–8	HPLC-FLD	[58]
AFs	Round fish tambaqui (*Colossoma macropomum*) pirapitinga (*Piaractus brachypomus*) pacu (*Piaractus mesopotamicus*)	Natural/fish farms	Muscle, (n = 26) liver (n = 26)	AFB1: 0.11–1.13, AFB2: 0.10–0.30, AFG1: 0.11–0.40, AFG2: n.d. AFB1: 0.15–5.7, AFB2: 0.78, AFG1: 0.18–0.27, AFG2: 0.16–0.38	HPLC-FLD	[53]
AFs, OTA	Rohu (*Labeo rohita*)	Natural/fish market	Muscle (n = 15), liver (n = 5)	AFs: 1.54, OTA: n.d. AFs: 3.86, OTA: 1.3	ELISA	[37]

n.d.—not detected; n—number of fish samples; AFs—Aflatoxins (B1, B2, G1, G2, M1); AFB1—Aflatoxin B1; OTA—Ochratoxin A; DON—Deoxynivalenol; T-2—T-2 toxin; HT-2—HT-2 toxin; ZEN—Zearalenone; STG—Sterigmatocystin; ELISA—enzyme-linked immunosorbent assay; LC-MS/MS—liquid-chromatography tandem mass spectrometry; HPLC-FLD—high-performance liquid chromatography with a fluorescence detector.

**Table 2:** Mycotoxin contamination in marine fish and derived products.

Mycotoxin	Fish Species	Feed Contamination/Fish Origin	Tissue/Organ n Fish	Contamination (µg/kg)	Detection Method	Reference
AFs, OTA	Sooly (*Lethrinus microdon*), Kurkufan (*Rhabdosargus haffara*)	Natural/fish from the market	Liver, muscle (n = 13)	AFs 1.08–1.93, OTA n.d.	ELISA	[37]
AFB1, T-2, OTA, DON	Large yellow croaker (*Pseudosciaena crocea*), Crimson snapper (*Lutjanus erythropterus*), Atlantic bluefin tuna (*Thunnus thynnus*), Japanese Spanish mackerel (*Scomberomorus niphonius*), Fourfinger threadfin (*Eleutheronema tetradactylum*), Largehead hairtail (*Trichiurus lepturus*)	Natural/fish from the market	Dried products (n = 70)	AFB1 0.03–3.52, T-2 0.21–1.53, OTA 0.03–2.21, DON 0.71	LC-MS/MS	[64]
OTA	European sea bass (*Dicentrarchus labrax*), Gilthead sea bream (*Sparus aurata*)	Natural/farmed fish	Muscle, liver, kidney (n = 60)	0.01–0.91	HPLC-FLD	[65]
AFB1, T-2, OTA, DON		Natural/fish from the market	Dried sea fish (n = 24)	DON n.d., T-2 0.61–1.07, AFB1 0.58–0.89, OTA 0.36–1.51	LC-MS/MS	[66]
ENNs, BEA	Gilthead sea bream (*Sparus aurata*), Atlantic salmon (*Salmo salar*)	Spiked/animal trial	Whole fish, fillet samples (n = 82)	n.d.	UHPLC-MS/MS	[61]
AFs, FUMs, ENNs, BEA, FUS-X, STG, OTA	Atlantic salmon (*Salmo salar*)	Natural/fish from the market	Products: smoked salmon, sushi salmon (n = 58)	n.d.	LC-MS/MS	[67]
AFs, ENNs, BEA, FUMs, STG, DON, 3AcDON, 15AcDON, NIV, NEO, DAS, FUS-X, ZEA, T-2, HT-2		Natural/fish from the market	Grilled salmon, grilled tuna, baked perch, grilled sole, cooked hake (unknown)	n.d.	LC-MS/MS	[68]
OTA, DON	Atlantic salmon (*Salmo salar*)	Spiked/animal trial	Plasma, liver, muscle, kidney, skin (n = 10 for each experiment)	DON 5.6–28.6 OTA 0.16–4.81	LC-MS/MS HPLC-FLD	[69]
ENNs, BEA	Atlantic salmon (*Salmo salar*) Gilthead sea bream (*Sparus aurata*) European sea bass (*Dicentrarchus labrax*)	Natural/fish from the market	Fillets (n = 30)	ENA1 1.7–29, ENB 1.3–103, ENB1 1.4–94, ENA n.d., BEA n.d.	LC-MS/MS	[70]
AFs, OTA, NEO, FUMs, T-2, HT-2, ZEN, NIV, DON, 3-AcDON, 15-AcDON, Fus-X	Gilthead sea bream (*Sparus aurata*), Atlantic salmon (*Salmo salar*)	Natural/Animal trial	Muscle (n = 6 for each treatment)	n.d.	UHPLC-MS/MS	[71]
AFs, OTA, ZEN, T-2, HT-2, DON		Natural/fish from the market	Dried sea fish products (n = 10)	OTA 1.9, ZEN 3.5–317.3	LC-MS/MS	[45]
ENNs, BEA	European sea bass (*Dicentrarchus labrax*), Sea bream (*Sparus aurata*)	Natural/fish from the market	Muscle, liver, head, viscera (n = 20)	BEA n.d., ENN 1.0–119.0	LC-MS/MS	[62]
ENNs, BEA	Sea bass (*Dicentrarchus labrax*), Gilthead sea bream (*Sparus aurata*), mackerel (*Scomber scombrus*), hake (*Merluccius merluccius*), and cod (*Gadus morhua*)	Natural/fish from the market	Whole fish (n = 19)	ENA n.d., BEA n.d., ENA1 1.51–7.45, ENB 1.30–44.65, ENB1 1.44–18.95,	LC_MS/MS	[63]
AFB1	European sea bass (*Dicentrarchus labrax*)	Spiked/animal trial	Muscle (n = 5 for each experiment)	0.29–4.25	unknown	[60]

n.d.—not detected; n—number of fish samples; AFs—Aflatoxins (B1, B2, G1, G2, M1); AFB1—Aflatoxin B1; OTA—Ochratoxin A; DON—Deoxynivalenol; T-2—T-2 toxin; HT-2—HT-2 toxin; ZEN—Zearalenone; FUMs (FB1–FB3)—Fumonisins; FUS-X—Fusarenon-X; NIV—Nivalenol; 3-AcDON—3-acetyldeoxynivalenol; 15-AcDON—15-acetyldeoxynivalenol; NEO—Neosolaniol; DAS—Diacetoxyscirpenol; ENNs (ENA, ENA1, ENB, ENB1)—Enniatins; BEA—Beauvericin; STG—Sterigmatocystin; ELISA—enzyme-linked immunosorbent assay; LC-MS/MS—liquid-chromatography tandem mass spectrometry; HPLC-FLD—high-performance liquid chromatography with a fluorescence detector.

## Data Availability

No new data were created or analysed in this study.
